# The Involvement of miR-29b-3p in Arterial Calcification by Targeting Matrix Metalloproteinase-2

**DOI:** 10.1155/2017/6713606

**Published:** 2017-01-09

**Authors:** Wenhong Jiang, Zhanman Zhang, Han Yang, Qiuning Lin, Chuangye Han, Xiao Qin

**Affiliations:** ^1^Department of Vascular Surgery, The First Affiliated Hospital of Guangxi Medical University, Nanning, Guangxi 530021, China; ^2^Department of Hepatobiliary Surgery, The First Affiliated Hospital of Guangxi Medical University, Nanning, Guangxi 530021, China

## Abstract

Vascular calcification is a risk predictor and common pathological change in cardiovascular diseases that are associated with elastin degradation and phenotypic transformation of vascular smooth muscle cells via gelatinase matrix metalloproteinase-2 (MMP2). However, the mechanisms involved in this process remain unclear. In this study, we investigated the relationships between miR-29b-3p and MMP2, to confirm miR-29b-3p-mediated MMP2 expression at the posttranscriptional level in arterial calcification. In male Sprague Dawley rats, arterial calcification was induced by subcutaneous injection of a toxic dose of cholecalciferol. In vivo, the quantitative real-time polymerase chain reaction (qRT-PCR) showed that MMP2 expression was upregulated in calcified arterial tissues, and miR-29b-3p expression was downregulated. There was a negative correlation between MMP2 mRNA expression and miR-29b-3p levels (*P* = 0.0014, *R*^2^ = 0.481). Western blotting showed that MMP2 expression was significantly increased in rats treated with cholecalciferol. In vitro, overexpression of miR-29b-3p led to decreased MMP2 expression in rat vascular smooth muscle cells, while downregulation of miR-29b-3p expression led to increased MMP2 expression. Moreover, the luciferase reporter assay confirmed that MMP2 is the direct target of miR-29b-3p. Together, our results demonstrated that a role of miR-29b-3p in vascular calcification involves targeting MMP2.

## 1. Introduction 

Arterial calcification increases morbidity and mortality in cardiovascular diseases. This process is also called ectopic or pathological mineralization of soft tissue and is considered a result of calcium phosphate crystal deposition on the vascular walls. This process can also be divided into two types based on the site of mineral deposits, including intimal calcification and medial calcification. The former, attributed to inflammation, oxidative stress, and disorders of lipid metabolism within the vascular walls, is common in atherosclerosis and coronary artery disease and contributes directly to vascular stenosis, myocardial ischemia, and infarction. The latter, named Monckeberg's medial sclerosis or elastocalcinosis, is a common pathological characteristic of vessels associated with aging, diabetes, renal failure, and vascular injuries. The two processes are followed by elastic fiber degradation and mineral deposits as linear deposits along the elastic lamellae, leading to arterial stiffening, hypertension, and myocardial hypertrophy [1–3]. Using different calcification models, researchers have recently conducted numerous studies involving the pathogenesis of vascular calcification. They have found that vascular calcification is a complex, active, cell-regulated process, which shares numerous similarities to physiological mineralization found in bones, teeth [4], and cartilage tissue, which have been detected by imaging [5]. Accumulating evidence has suggested that vascular calcification is related to transdifferentiation to a chondrocyte or osteoblast-like phenotype of vascular smooth muscle cells (VSMCs) [6, 7] and matrix vesicle or apoptotic bodies, which provides binding sites for calcium phosphate [8, 9]. Elastin degradation, which has a high affinity for calcium [10], can induce a osteogenic response in VSMCs [11, 12]. Previous studies have provided abundant information about the pathogenesis of vessel calcification, but the precise mechanisms have yet to be fully characterized.

Matrix metalloproteinases-2 (MMP2), a gelatinase that has an important role in matrix degradation and vascular remodeling, is involved in many cardiovascular diseases, including aortic aneurysm formation, atherosclerosis, and myocardial fibrosis [13]. Upregulation of MMP2 expression and activity have been reported in VSMCs and animal calcification models, which mediate elastin degradation, producing soluble elastin peptides and causing release and activation of the latent TGF-*β*1 complex from the extracellular matrix of vessel walls. In cultured rat VSMCs [14], a soluble elastin peptide upregulated the expression of typical bone proteins with synergism of TGF-*β*1 via elastin laminin receptor signaling [15–17]. Furthermore, TGF-*β*1 and its mediated signaling pathway have important roles in physiological and vascular calcification [18]. MMP2 upregulation contributes to bone morphogenetic protein 2 (BMP2) expression in a *β*-glycerophosphate-induced VSMC calcification model [19] that could increase* RUNX* and* MSX2* expression and promote VSMC calcification [20]. The regulation of MMP2 expression is therefore associated with the prevention and treatment of vascular calcification. However, few studies have reported that posttranscriptional regulation of MMP2 is involved in vascular calcification.

In recent years, microRNAs, a novel class of endogenous, small, single-stranded noncoding RNAs that mediate epigenetic regulation, have been shown to play a role in gene expression in many diseases. The microRNAs induce specific mRNA degradation or block translation by binding to the 3′-UTR of the targeting mRNA [21]. In vascular biology, microRNA dysregulation was found to contribute to various pathological conditions (atherosclerosis and a proliferative thickening and restenosis after vessel injury) through the modification of vessel cell functions [22, 23]. There has been evidence that specific miRNAs also play critical roles in the regulation of vascular calcification, both in vitro and in vivo. Osteoblast-like phenotypic VSMC and elastin degradation play central roles in vessel calcification. Gain- and loss-of-function miRNA studies and luciferase reporter assays reported that miR-204 [24], miR-133a [25], and miR-30b-c [26] inhibited the osteogenic differentiation of mice VSMCs and human coronary artery smooth muscle cells (SMCs) during *β*-glycerophosphate-induced calcification through the targeting of RUNX2. However, miR-mediated MMP expression in arterial calcification needs to be further characterized.

The miR-29 family (miR-29a, miR-29b-3p, and miR-29c) has been previously implicated in multiple pathological changes in cardiovascular diseases. It has been reported that peripheral plasma levels of miR-29a in hypertrophic cardiomyopathy patients were significantly upregulated, when compared with a control group, and these levels correlated with both hypertrophy and fibrosis [27]. Furthermore, all members of the miR-29 family were downregulated in myocardial tissue adjacent to the infarct and during cardiac fibrosis after acute myocardial infarction in mice and humans [28]. In addition, accumulating evidence suggests that miR-29 downregulation inhibits dilation of aneurysms and is helpful in an early fibrotic response at the aortic wall by increasing target gene expression in murine models of experimental aneurysms and human aneurysm tissues [29]. In recent years, some studies have reported that miR-29 is a novel diagnostic biomarker and therapeutic target for cancer, because it plays an important role in angiogenesis, invasion, and metastasis of tumors by regulating MMP2 expression [30].

In this study, we elucidated the relationships between MMP2 and miR-29b-3p during arterial calcification and characterized the effects and mechanisms for regulation of calcification in vitro and in vivo. Taken together, these results provide a candidate molecule and model for therapeutic intervention of vascular calcification.

## 2. Materials and Methods 

### 2.1. Ethics Statement

All animal studies were approved by the Ethics Committee of Guangxi Medical University (Approval Number: 201511018). The studies conformed with the guide for the care and use of laboratory animals published by the China National Institutes of Health.

### 2.2. Rat Thoracic Aorta Calcification Model

Male Sprague Dawley rats, which were from the experimental animal center of Guangxi Medical University, with a mean body weight of 150–200 g, were used for the studies. The animals had free access to water and food. After an acclimatization period of 2 days, the animals were randomly allocated to experimental groups. The rat thoracic aorta calcification model was established as previously described [31, 32]. Rats were killed 7 days after the first vitamin D3 injection by cervical dislocation while they were under metofane-induced anesthesia. The aortic tissues between the aortic arch and 1 cm below the renal branch were immediately removed after death. The tissues of the thoracic aorta were fixed in 4% paraformaldehyde for at least 24 h at room temperature. Sectioning and histological staining (HE and Von Kossa staining) of the paraformaldehyde-fixed tissues were performed for histological analyses of mineral accumulation and for assessment of success in creating a rat thoracic aorta calcification model. The other aortic tissues were immediately frozen in liquid nitrogen until they were analyzed for RNA and protein content.

### 2.3. Von Kossa and HE Staining

Paraffin blocks of aortic tissue were cut into 8 um sections and subjected to Von Kossa staining by following the protocol of the Von Kossa staining kit (Jianchengbio, Nanjing, China). In brief, the slides were deparaffinized, hydrated with distilled water, and incubated in 5% silver nitrate solution for 1 h under a strong sunlight. The silver nitrate solution was then diluted with distilled water and the sections were incubated with a 5% thiosulphate solution for 10 min. Other sections were stained with HE. Images were captured using a light microscope, and calcium phosphate deposits were detected as black-stained areas.

### 2.4. Cell Culturing and Transfection

VSMC lines (A7R5; ATCC CRL-1444) (Aolu Biotech, Shanghai, China) were cultured in a humidified atmosphere at 37°C and 5% CO_2_. Standard culture medium consisted of Dulbecco's Modified Eagle Medium (DMEM) with 50 *μ*g/mL streptomycin and 50 units/mL penicillin and was supplemented with 10% fetal bovine serum (FBS) (Grand Island Biological, Grand Island, NY, USA). Cell layers were detached with 0.25% trypsin/0.02% EDTA solution (Solarbio, Beijing, China). Cells were maintained at 80%–90% confluency by passaging as needed. A7R5 VSMCs were used from passages 5–10.

Mimics (5′-UUGUGACUAAAGUUUACCACGAU-3′) and inhibitors of rno-miR-29b-3p (5′-AACACUGAUUUCAAAUGGUGCUA-3′) and the negative control (5′-UCACAACCUCCUAGAAAGAGUAGA-3′) were transfected into A7R5 VSMCs. The transfection used a CP transfection kit (Ribobio, Guangzhou, China), according to the manufacturer's protocol. The cells were plated in six-well plates with DMEM supplemented with 10% FBS at a density of 1 × 105 cells per well 24 h prior to the transfection. For expression, miR-29b-3p and mimics of miR-29b-3p were transfected into cells at a final concentration of 50 nm. After transfection for 48 h, RNA was extracted using TRIzol® (Invitrogen, Carlsbad, CA, USA), and successful transfection was confirmed by the quantitative real-time polymerase chain reaction (qRT-PCR) for miR-29b-3p. Random sequence miR was used as a negative control. To inhibit miR-29b-3p expression, cells were transfected with 150 nm inhibitors of miR-29b-3p using a similar method as described for the transfection of mimics.

### 2.5. qRT-PCR for MMP2 and rno-miR-29b-3p

Total RNA containing the miRs was isolated from cells and frozen aorta tissues using TRIzol, according to the manufacturer's instructions (Invitrogen). For each sample, the total RNA content was determined by measuring the absorbance at 260 nm and the purity was determined using* A*260/*A*280 ratios. For qRT-PCR, the first strand cDNA of miRNA and mRNA was reverse transcribed from total RNA using the Mir-XTM miRNA first strand synthesis kit and PrimeScript™ RT reagent kit with gDNA eraser (Takara Biotechnology, Dalian, China), respectively, and 2 *μ*L of the first strand cDNA was used for detecting mRNA and miRNA expression using the real-time polymerase chain reaction (RT-PCR) (ABI 7500 Fast; Applied Biosystems, Darmstadt, Germany) using the FastStart SYBR Green Kit (Roche, East Sussex, UK). The specific PCR primers used were rat MMP2 (forward primer, 5′-ACCTTGACCAGAACACCATCGAG-3′, reverse primer, 5′-CAGGGTCCAGGTCAGGTGTGTA-3′) and rat *β*-actin (forward primer, 5′-GGAGATTACTGCCCTGGCTCCTA-3′, reverse primer, 5′-GACTCATCGTACTCCTGCTTGCTG-3′). The rat U6 primer and miR-29b-3p were purchased from Genecopoeia (Guangzhou, China). Expression of MP2 and miR-29b-3p were normalized with *β*-actin and U6, respectively, and the relative expression was determined by the comparative CT (2^−ΔΔCt^) method.

### 2.6. Western Blot Analysis

To investigate rat aorta and cellular MMP2 protein expression, western blotting was performed according to the Abcam western blot instructions. Western blots were performed with 50 *μ*g of total protein. The antibodies used were anti-MMP2 (ab37150; Abcam, Cambridge, MA, USA) and anti-actin (KC-5A08, Kang Cheng Bio-tech, Shanghai, China). Image-Pro Plus 6.0 software (Media Cybernetics lnc., Maryland, USA) was used for analyzing the results of densitometric scans of the bands. The results were normalized to total protein levels and expressed as a ratio of *β*-actin.

### 2.7. Luciferase Reporter Assay

The public databases (TargetScan http://www.targetScan.org and mirbase http://www.mirbase.org/) were searched for predicting the miRNA for regulating rat MMP2. To confirm the regulation between miR-29b-3p and MMP2, a luciferase reporter assay was performed in HEK293T cells. The cells were grown to 60% confluency in 24-well plates in complete culture medium [high glucose DMEM (Gibco, New York, USA) with 10% FBS, 50 *μ*g/mL streptomycin, and 50 units/mL penicillin] with luciferase reporter plasmids carrying wild-type MMP2 3′-UTR, mutant MMP2 3′-UTR, and a 3′-UTR negative control and rno-miR-29b-3p or rno-miR negative control plasmid and renilla plasmid cotransfected into HEK 293T cells. For assessing the efficacy of the transfection system, the TRAF6 3′-UTR plasmid and the rno-mir negative control plasmid or has-miR-146b plasmid were also cotransfected into cells. Cells were collected 48 h after transfection and analyzed using the Luciferase Assay System, according to the manufacturer's instructions (Promega Corporation, Madison, Wisconsin, USA).

### 2.8. Statistical Analyses

SPSS statistical software for Windows, version 20.0 (SPSS, Chicago, IL, USA), was used for all analyses. Data were expressed as the mean ± SD. Comparisons were made using a two-sample test in two groups, and one-way ANOVA was used for multiple comparisons. The relationship between rno-miR-29b-3p and MMP2 mRNA and protein was determined using linear least squares regression analyses. The results were reported graphically and a correlation constant (*R*^2^) and a probability value. *P* < 0.05 was considered statistically significant.

## 3. Results 

### 3.1. Establishment of a Thoracic Aortic Calcification Model

**Figure 1:**
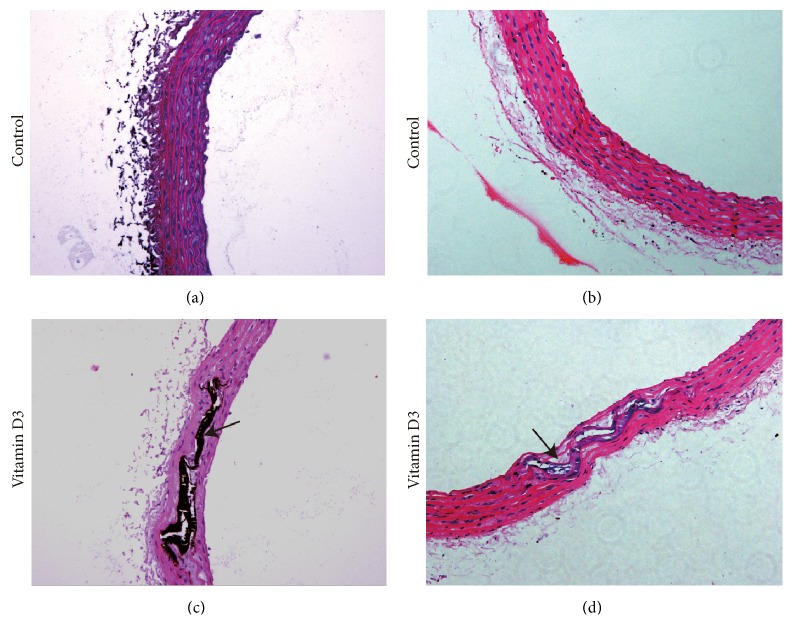
Von Kossa and hematoxylin and eosin (HE) staining for aortic sections from the rat. (a) Von Kossa staining for aortic sections in the control group. (b) HE staining for aortic sections in the control group. (c) Von Kossa staining for aortic sections in the vitamin D3 group. (d) HE staining for aortic sections in the vitamin D3 group. The black arrow designates excessive mineral deposits.

Von Kossa staining and hematoxylin and eosin (HE) staining showed extensive mineral deposition, elastic fibers, and extracellular matrix degradation in aorta media in the group receiving cholecalciferol. These pathological changes were not found in structural features of the vascular walls in the control group (Figure 1). The results suggested that the vascular calcification model was successfully induced in rats after injection with cholecalciferol.

### 3.2. MMP2 and miR-29b-3p Are Involved in Arterial Calcification

**Figure 2:**
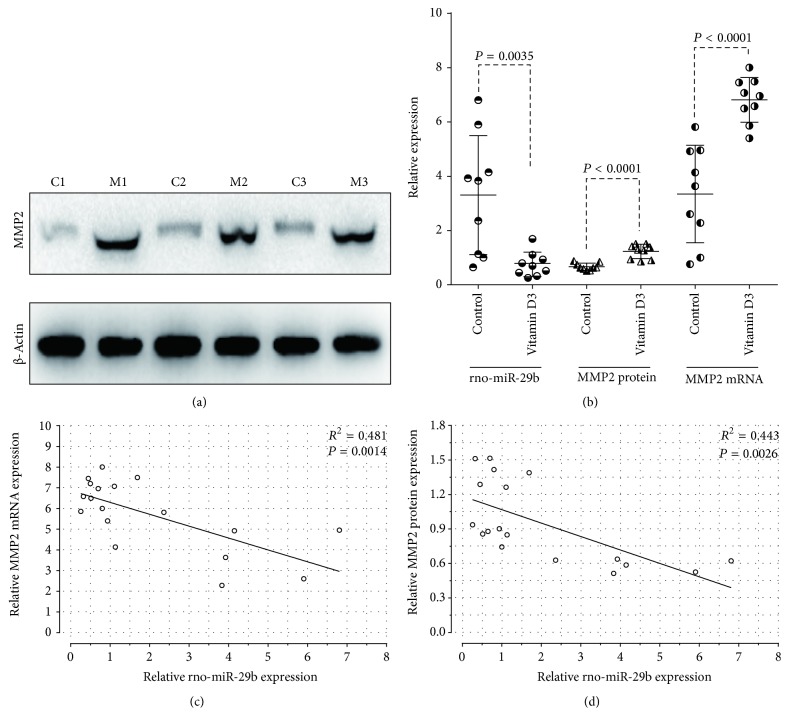
Analyses of MMP2 and miR-29b-3p expression in the vitamin D3 group and the control group. (a) Western blot analyses for MMP2 protein in rat aortas from the control group (C1, C2, and C3) and the vitamin D3-treated group (M1, M2, and M3). (b) Scatter diagram for expression of MMP2 and miR-29b-3p. MMP2 expression was significantly increased and miR-29b-3p was significantly downregulated in the vitamin D3 group. (c, d) Correlation analyses to determine the relationships between miR-29b-3p and MMP2 mRNA levels and MMP2 protein in the control and vitamin D3 groups (MMP2 mRNA versus miR-29b-3p, *R*^2^ = 0.481, *P* = 0.0014; MMP2 protein versus miR-29b-3p, *R*^2^ = 0.443, *P* = 0.0026).

MMP2 and miR-29b-3p are associated with vascular calcification induced by cholecalciferol in rats. Compared with the control group, the expression of MMP2 mRNA and protein were upregulated in the cholecalciferol treatment group (both, *P* < 0.0001; Figure 2). However, in the calcification rat group, miR-29b-3p was significantly decreased (*P* = 0.0035). Moreover, we investigated whether MMP2 expression correlated with miR-29b-3p levels, which may be involved in regulating target gene expression at the posttranscriptional level. Correlation analyses showed that there was a negative correlation between MMP2 protein levels and mRNA and miR-29b-3p expression (*R*^2^ = 0.443 and 0.481, resp.; *P* = 0.0026 and 0.0014, resp.), as shown in Figure 2. These data suggested that miR-29 may be involved in vascular calcification through mediation of MMP2 expression.

### 3.3. The Effect of Inhibition and Overexpression of miR-29b-3p on MMP2 Expression in Rat VSMCs

**Figure 3:**
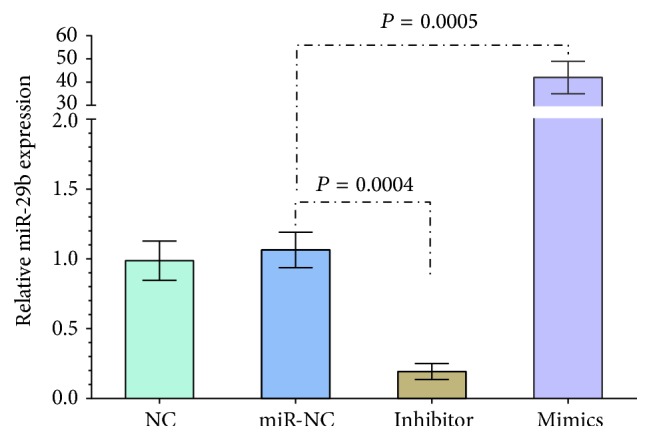
Transfection of miR-29b-3p mimics into vascular smooth muscle cells increases the miR-29b-3p expression, and the inhibitor downregulates miR-29b-3p expression, as determined by the quantitative real-time polymerase chain reaction. Data are expressed as the mean ± SD, and each experiment was repeated three times.

**Figure 4:**
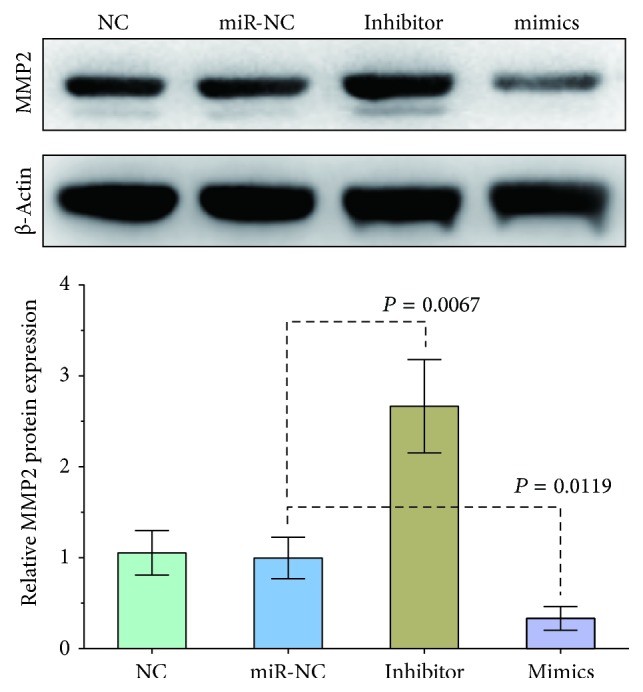
The effect of inhibition and overexpression of miR-29b-3p on MMP2 expression in rat vascular smooth muscle cells. The miR-29b-3p mimics and inhibitors regulate MMP2 expression as determined by western blot analyses.

To further characterize the relationship between miR-29b-3p and MMP2, we used bioinformatics and a target prediction database of miRs, to determine that MMP2 was a potential target of miR-29b-3p. Furthermore, exogenous transfection of miRs was used to determine whether miR-29b-3p can regulate MMP2 expression in rat VSMCs. The mimic, inhibitor, and control sequences of miR-29b-3p were transfected in VSMCs to change the levels of cellular miR-29b-3p (Figure 3). The results of this transfection showed that an increase of miR-29b-3p inhibited MMP2 expression and loss of miR-29b-3p-induced MMP2 expression in rat VSMCs, indicating that miR-29b-3p had an effect on MMP2 expression, either directly or indirectly (Figure 4).

### 3.4. miR-29b-3p Directly Regulates MMP2 Expression

**Figure 5:**
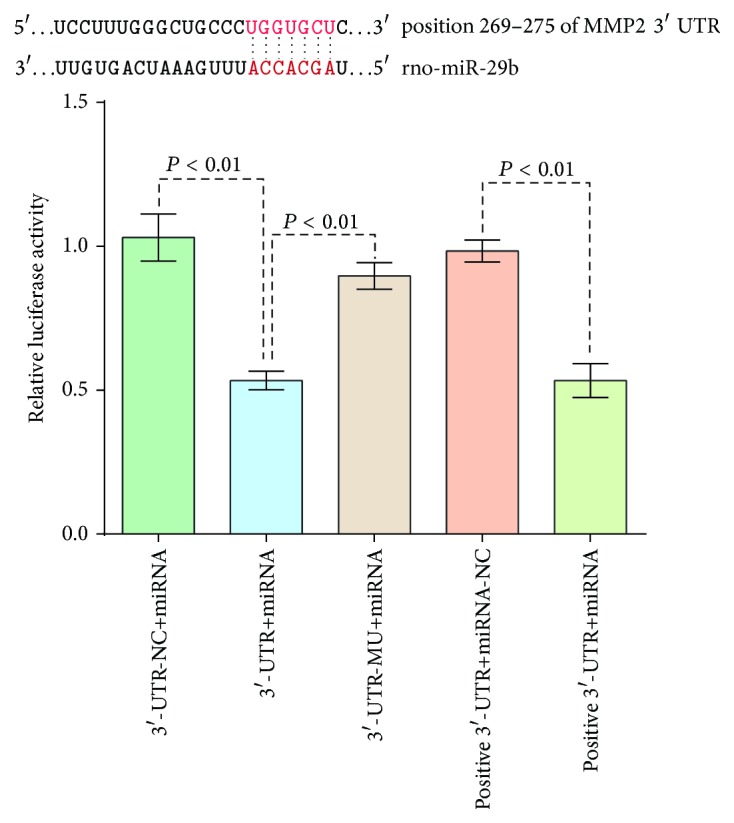
Target verification between miR-29b-3p and MMP2 in HEK293T cells. The luciferase reporter plasmids (3′-UTR wild-type, 3′-UTR mutant of MMP2, and the 3′-UTR negative control) and the renilla plasmid were cotransfected into HEK293T cells. The TRAF6 and has-miR-146b genes were used for the positive control group. Data was expressed as the mean ± SD, and each experiment was repeated three times.

To further characterize the mechanisms involving miR-29b-3p-mediated MMP2 expression, a luciferase assay was performed to determine whether miR-29 can directly target MMP2.  Figure 5 shows that seven bases of rno-miR-29b-3p in the seed region are complementary with MMP2 3′-UTR bases at positions 269–275. The relative luciferase activity was significantly inhibited by overexpression of miR-29b-3p in the wild-type 3′-UTR of the MMP2 group compared with the MMP2 3′-UTR-negative control group (*P* < 0.001) and MMP2 3′-UTR-mutant group (*P* < 0.001). In addition, gain of position miR (has-miR-146b) decreased the relative luciferase activity in the 3′-UTR (TRAF6) group, confirming that the transfection system was functional. These results indicated that MMP2 is a direct target gene of miR-29b-3p.

## 4. Discussion 

In the present study, we showed that the expression of miR-29b-3p was downregulated in rat models of vascular calcification induced with cholecalciferol when compared with the control group. Furthermore, there was a negative correlation between miR-29b-3p expression and MMP2 expression.

Overexpression of miR-29b-3p or inhibition of miR-29b-3p in rat VSMCs resulted in MMP2 downregulation or upregulation at the protein level, respectively. Moreover, a luciferase reporter assay was used to show that miR-29b-3p directly inhibited MMP2 expression. Taken together, the results showed that miR-29b-3p was involved in vascular calcification via targeting of MMP2 expression.

It is well-known that MMP2 plays a pivotal role in the pathogenesis of vascular calcification involved in elastin degradation and VSMC transdifferentiation to an osteoblast-like phenotype [19, 33–35]. Inhibition or knock-down of MMP2 in vitro and in vivo decreased arterial and VSMCs calcification in multiple calcification models [11, 12, 36–40]. Similarly, in our study, miR-29b-3p was downregulated during rat calcification compared with the control group, implying that miR-29b-3p inhibited vascular calcification by some unknown mechanism. Notably, upregulation of MMP2 negatively correlated with miR-29b-3p in calcified arterial tissues. We therefore speculate that miR-29b-3p plays a role in MMP2 expression and is involved in vascular calcification. Subsequent studies reported that miR-29b-3p directly regulated MMP2 expression, as shown by overexpression and inhibition of miR-29b-3p with mimics, inhibitors, and a luciferase reporter assay. The relationship between miR-29b-3p and MMP2 was consistent with our finding that miR-29b-3p downregulation resulted in MMP2 upregulation and promoted vascular calcification in rats.

It has been generally accepted that miRs are involved in the pathology of many cardiovascular diseases through their expression regulation of different target genes [41]. The rat miR-29 family includes three members: miR-29a, miR-29b-3p, and miR-29c, whose dysregulation has been reported in many studies. Some studies indirectly supported our results, although there were some differences in some of the studies. Our finding that miR-29b-3p was downregulated in vascular calcification was consistent with the results of Du et al. [42], who reported that miR-29b-3p inhibits vascular smooth muscle cell calcification by decreasing the target genes disintegrin and metalloproteinase with thrombospondin motif-7 (ADAMTS-7) expression, in an inorganic phosphorus and chronic kidney disease-induced rat calcification model. The difference of miR-29b-3p target genes in the two studies can be explained by the observation that specific miRNAs may target multiple mRNAs and that a specific mRNA may contain binding sites for different miRNAs. In addition, the miR-29 family is involved in aneurysm development by mediating extracellular matrix protein synthesis, including Col1a1, Col3a1, Col5a1, and Eln. Maegdefessel et al. [29] reported that miR-29b-3p downregulation significantly inhibited abdominal aortic aneurysm (AAA) expansion and progression and promoted fibrosis within AAA walls to decrease the aneurysm rupture rate in two mice AAA models induced with porcine pancreatic elastase and angiotensin II (angII). Similar results were observed in human thoracic aneurysm tissue. It has been reported that MMPs synthesis and activity are increased in rat and human aneurysm tissue, especially MMP2 and MMP9, which can degrade elastin, resulting in less flexibility and loss of the integrity of the vessel walls. Elastic fiber degradation is the earliest step in medial calcification in chronic kidney disease and diabetic patients. Surprisingly, there was also a negative correlation between miR-29a and total MMP2 expression in human thoracic aortic aneurysm tissue [43]. Furthermore, miR-29b-3p largely decreased, resulting in myocardial fibrosis of angiotensin II, involved in the TGF-*β*1/Smad signal pathway in the angII-induced hypertensive heart disease mouse model [44]. However, many studies have reported that increasing angII can induce MMP2 upregulation and activity, which activates TGF-*β*1/Smad signaling, and is associated with a central arterial wall stiffness, such as fibrosis and vascular calcification [45]. We therefore speculate that there is an opposite trend among expressions of miR-29b-3p, MMP2, TGF-beta1, and miR-29b-3p downregulation, contributing to vascular calcification by upregulation of MMP2 and TGF-beta1 in the angII-induced calcification model. Recently, mcl, an antiapoptotic Bcl-2 family member, has been also reported to be a target of miR-29b-3p in HeLa and KMCH cells [46], and miR-29b-3p downregulation inhibited apoptosis and promoted cancer cell proliferation. Apoptotic bodies from cell apoptosis provide binding sites for minerals involved in the first step of vascular calcification [47]. The results also suggested that miR-29b-3p can negatively regulate vascular calcification. Moreover, in the chronic kidney disease mice, miR-29a/b was significantly suppressed in muscle [48]. However, medial elastin-specific calcification is a prevalent complication in patients and animal models with chronic kidney disease.

Although our results are consistent with other studies, there is still controversy. It has been reported that increased miR-29b-3p expression promoted VSMC calcification by decreasing elastin expression in the inorganic phosphorus-induced VSMC calcification model [49]. These differences with our study results can be explained in two ways. First, calcification models can be induced in different ways that involve different mechanisms. Many possible mechanisms have not been identified in vitamin D3 intoxication-induced arterial calcification. Nonetheless, this process can stimulate bone resorption and elevate serum calcium, which play important roles in VSMC apoptosis and vesicle release, while increased inorganic phosphorus has a major role in osteogenic/chondrogenic differentiation of VSMCs [50]. Second, we induced the aortic calcification model in rats in vivo, which was regulated by different factors compared with VSMC calcification in vitro. These findings are indicative of the complex mechanism of gene regulation.

Our study had some limitations. First, the levels of pre-mRNA for MMP2 were not measured in our studies. The transcriptional activity of a MMP2 decrease may have a role for MMP2 downregulation at mRNA and proteins levels in the cholecalciferol-induced rat calcification model, but it does not affect the miR-29b-3p inhibition of MMP2 expression at posttranscriptional levels, as shown by transfecting with miR-29b-3p mimics and inhibitors and the luciferase reporter assay. Furthermore, whether miR-29b-3p is a treatment for vascular calcification in vivo and in vitro needs further studies. In future studies, we plan to overexpress and knock-out miR-29b-3p with lentivirus in VSMCs and then test whether miR-29b-3p plays a role in potential mechanisms of vascular calcification.

## 5. Conclusion 

Our results indicated that miR-29b-3p is involved in the progression of pathological vascular calcification by targeting MMP2. This process may be a treatment option for vascular calcification. However, whether miR-29b-3p can be used as a treatment for vascular calcification both in vivo and in vitro needs to be confirmed with further studies.

## Abbreviations

MMP2: Matrix metalloproteinase-2
qRT-PCR: Quantitative real-time polymerase chain reaction
VSMCs: Vascular smooth muscle cells
AAA: Abdominal aortic aneurysm.
